# Toward an improved in vitro model of prosthetic joint infection for *Staphylococcus aureus* biofilm characterization

**DOI:** 10.1016/j.bioflm.2025.100325

**Published:** 2025-10-08

**Authors:** Yasser Dghoughi, Jennifer Varin-Simon, Sophie C. Gangloff, Marius Colin, Fany Reffuveille

**Affiliations:** a Université de Reims Champagne-Ardenne, URBIOS, Reims, France; b Université de Reims Champagne-Ardenne, UFR de Pharmacie, Reims, France

**Keywords:** *Staphylococcus aureus*, Biofilms, Periprosthetic joint infections, *In vitro* model

## Abstract

Biofilm formation on orthopedic implants is often implicated in chronic prosthetic and joint infections (PJI) that are complex to manage. To date, no current bacterial *in vitro* model can fully simulate the PJI environment leading to a lack of knowledge to develop diagnosis tool and adapted treatment. Our project aims to set up an innovative *in vitro* model to characterize *Staphylococcus aureus* clinical strains biofilms in a PJI context, focusing on several parameters: culture media, incubation time, atmospheric conditions and support for biofilm growth.

Biofilm formation was evaluated in various culture media, by counting both planktonic and adherent bacteria (CFU) and quantifying biofilm biomass using crystal violet staining. A mature biofilm was obtained after 72 h of incubation with a similar proportion of planktonic and adherent bacteria whereas a variable dispersion was observed at 96 h.

Comparing two different oxygen concentrations (Hypoxia 2.5 % like in bone site *vs* Anoxia) revealed that a slight variation had a strong impact on biofilm formation, underlining the fact that the physiological conditions are highly necessary to set a mimetic model. A medium has therefore been developed, the modified Bone-Like Environment (BLE+) allowing a consistent biofilm growth.

When studying bacterial adhesion, planktonic bacteria can gather and form aggregates that are distinct from mature biofilms. To avoid this phenomenon, a suspended pegs was used. By holding the pegs in the medium, we specifically drove active bacterial adhesion related to biofilm formation, eliminating interference from sedimented aggregates. Moreover, to limit the interaction between planktonic bacteria and biofilm over the 72 h, a medium renewal was applied at 8 h of incubation with a low impact on biofilm biomass.

This method allowed the observation of differences between the USA300 (MRSA) and SH1000 (MSSA) strains: the MSSA showed more adherent bacteria and bigger aggregates than the MRSA strain.

In conclusion, the parameters for an *in vitro* biofilm model simulating PJI context have been validated. These parameters include 2.5 % dioxygen, BLE + supplementation, and 72-h incubation on suspended titanium pegs with a renewal media after a primo bacteria adhesion of 8 h.

## 1 Introduction

In nature and clinical settings, biofilms emerge as sophisticated microbial structures that allow bacterial survival, persistence, and pathogenicity. Ubiquitous in the natural environment, biofilms constitute communities of adhered microorganisms distinct from planktonic bacteria, which exist as free-floating individual entities [1]. Formed by bacteria gathering together and adhering to biotic or abiotic surfaces [2], biofilms are also made up of a complex extracellular matrix (ECM) composed of polysaccharides (notably Polysaccharide Intracellular Adhesin (PIA) and Poly-*N*-Acetylglucosamnie (PNAG)), extracellular DNA, and adhesive proteins (e.g., Fibronectin-Binding Protein (FnBPs)) [1]. Altogether these components, provide structural stability and mediate surface attachment, creating a microenvironment often containing essential nutrients and oxygen. Crucially, this matrix shelters bacteria against host defenses (both innate and adaptive immunity) and antibiotic agents [3,4].

Biofilms are involved in various diseases including wounds, urinary tract infections, and prosthetic joint infections (PJIs) and may contribute indirectly to the development of several cancers [5]. These biofilm-associated diseases pose significant health threats due to their resilience against conventional treatments which favors their chronicity [5]. Biofilms are the primary drivers of implant-associated infections, a concern exacerbated by the rising incidence of prosthetic joint replacements, driven by the expansion of elderly population [6].

Whether the infection arises from endogenous bacteria from the patient flora or exogenous sources, *Staphylococcus aureus* is detected in over 30 % of PJIs, establishing it as the most prevalent species [7]. Recognized by the World Health Organization (WHO) as a highly virulent bacterial species among “ESKAPE” (*Escherichia coli, Staphylococcus* spp*, Klebsiella pneumoniae, Acinetobacter baumannii, Pseudomonas aeruginosa* and *Enterococcus* spp.*)* pathogens [8], diagnosis of *S. aureus* biofilm infections remains a significant challenge, even when clinical signs, such as erythema and swelling, are strong [9]). Reaction to a foreign body and infection can both trigger a similar inflammatory response, making it difficult to clinically distinguish them [[9], [10], [11]]. Moreover, phenotypic heterogeneity within biofilms allows some bacteria to enter dormant states, making them hardly detectable by conventional diagnostic techniques [12]. Additionally, the bacterial attachment complicates removal from tissue surfaces and implant materials [12]. These challenges can lead to false-negative results in diagnostic tests, delaying the initiation of adapted therapy.

Additionally, antibiotics are often ineffective against biofilms. Even worse, the overuse of antibiotics leads strains to develop tolerance to them, which can lead to resistance acquisition and transfer [11,12].

The behavior of *S. aureus* biofilms varies significantly across strains, influencing the severity of infections and treatment strategies. Despite recent improvements in understanding biofilm, clinical success in eradicating PJI through revision surgery remains challenging, around 20 % of cases requiring lifelong suppressive antibiotic treatment and/or reoperation [13,14]. Although this therapeutic approach is carried out in the early stages of the infection, the difficulty to access to the infectious site by the antibacterial agent, coupled in some cases with a long course of antibiotics [15], leads irremediably to the appearance of persistence mechanisms, especially biofilm formation. Based on the literature [16] environmental factors such as temperature, pH, salt or glucose concentrations significantly influence biofilm development [17]. Indeed, biofilms vary depending on the environment, leading to the necessity to study infectious biofilms in the appropriate context [18].

Thus, while biofilm plays a pivotal role in PJI, current diagnostic techniques are poorly adapted to biofilm evaluation. Standard diagnostic methods like Minimum Inhibitory Concentration (MIC) and Minimum Bactericidal Concentration (MBC) are designed for planktonic bacteria, overlooking the biofilm complex architecture and resilience [19,20]. When antibiofilm candidates are tested under conditions that do not accurately mimic infectious sites, their efficacy may be overstated, resulting in limited effectiveness *in vivo* [21]. For example, simplified static models are often used, along with inappropriate media, typically rich media, that lack the physiological and immunological factors present in actual infection sites [22,23]. Although sophisticated techniques exist to analyze biofilms, including those targeting specific biofilm-associated proteins (Confocal microscopy with fluorescent labelling, Transcriptomics/proteomics …), resource limitations often lead researchers to rely on simplified analysis [24,25].

The Calgary Biofilm Device (CBD) presents a more biofilm-focused approach. Developed in the early 2000s, CBD expedites the assessment of antibiofilm effects by using plastic pegs affixed to lids, promoting biofilm formation on the pegs while planktonic cells are developing in the media. Biofilms can then be studied directly on the pegs in the case of biomass investigation, or through bacteria resuspension via sonication. However, some important challenges are still remaining: (*i*) the use of plastic surfaces is still far from the clinical environment which can highly influence the adherence of bacteria and more generally the biofilm behavior; (*ii*) they do not allow easy microscopic observation of bacteria adhered to the pegs (fixed to the lid). This limitation restricts the ability to analyze biofilm formation, structure and composition.

All model limitations highlight the need for advanced, physiologically relevant, and easy-to-handle models to enhance diagnostic accuracy and therapeutic strategies. Therefore, there is an urgent need to move beyond conventional study models and adopt innovative approaches that more closely mimic the physiological context.

In this study, we have sought to develop a new *in vitro* model that parts away from traditional methods, moving away from the plastic model, and distinguishing itself from conventional media by moving closer to the bone prosthesis environment. We aimed to find a model that would balance user-friendliness and accuracy. To characterize the model, we focused on developing a system that allows the quantification of bacterial biomass, the assessment of viable bacteria, and the analysis of biofilm composition and its structure over time.

## 2 Materials and methods

### 2.1 Media preparation

.

**Table 1:** Components for media preparation: KH_2_ from Fisher Scientific, PO_4_ from Fisher Scientific, (NH_4_)_2_SO_4_ from Fisher Scientific, MgSO_4_ from ACROS Organics, FeSO_4_ from ACROS Oganics, Glucose from Fisher Scientific, Casamino Acids from Becton Dickinson (BD).

Media (Right)	Minimum Media (MM)	Bone-Like Environment (BLE)	Modified Bone-Like Environment (BLE+)
Components (Down)			
**KH_2_PO_4_**	**62 mM**	**62 mM**	**62 mM**
**(NH_4_)_2_SO_4_**	**7 mM**	**7 mM**	**7 mM**
**MgSO_4_**	**2 mM**	**20 mM**	**20 mM**
**FeSO_4_**	**10 μM**	**10 mM**	**10 mM**
**Glucose 20 %**	**4.44 mM**	**0**	**888 μM**
**Casamino Acids (CAA) 5 %**	**181 mM**	**0**	**36.3 mM**

### 2.2 Bacterial strains and culture media

Two *Staphylococcus aureus* strains were used: SH1000 and USA3000. SH1000 is a methicillin-sensitive strain and originated from 8325 to 4 strain with repaired *rsbU* gene (Horsburgh et al., 2002). USA300 is a methicillin-resistant strain USA300 discovered in the 1990s in the United States with staphylococcal cassette chromosome *mec IVa*, a Methicillin resistance cassette and arginine catabolic mobile element (ACME) a mobile element promoting skin colonization.

Shortly after isolating the strain on a tryptic soy agar (TSA) plates (*Biokar diagnostics®*, Pantin, France), a single colony is transferred into 3 mL of nutrient broth (Bio-Rad, Marnes-la-Coquette, France), vortexed, and then incubated overnight at 37 °C. The absorbance of overnight cultures was measured at 600 nm and the culture was then diluted in appropriate fresh medium (MM, BLE, BLE +) (Table I) to obtain an inoculum with an estimated final absorbance of 0.1 (An initial count was carried out to confirm the concentration of bacteria inoculated.). Media containing bacteria were deposited in microtiter plates (24 well-plates for disk and 96-wellplates for peg) and they were incubated at 37 °C for 24 h, 72 h, 96 h or 144 h. Experiments under hypoxic conditions were performed using an incubator set on 2.5 % of O_2_. For experiments in anoxic conditions, the GenBag system (Biomérieux, Marcy-l’Étoile, France) was used according to the manufacturers protocol.

**Fig. 1:**
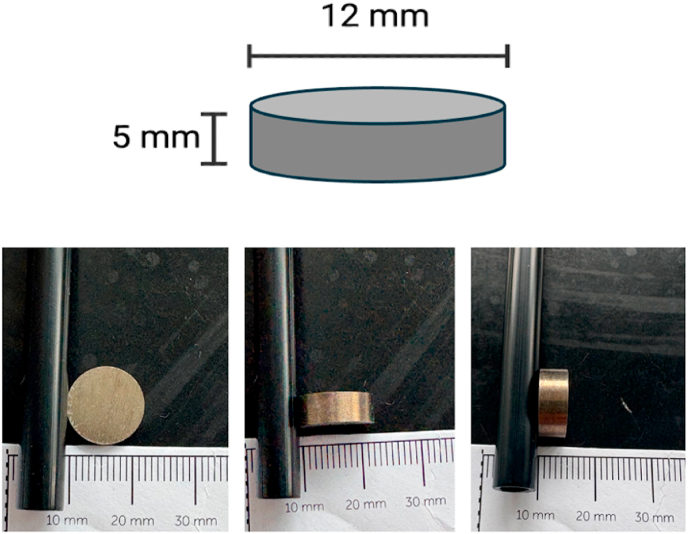
Figure and dimensions of disc used in the model.

**Fig. 2:**
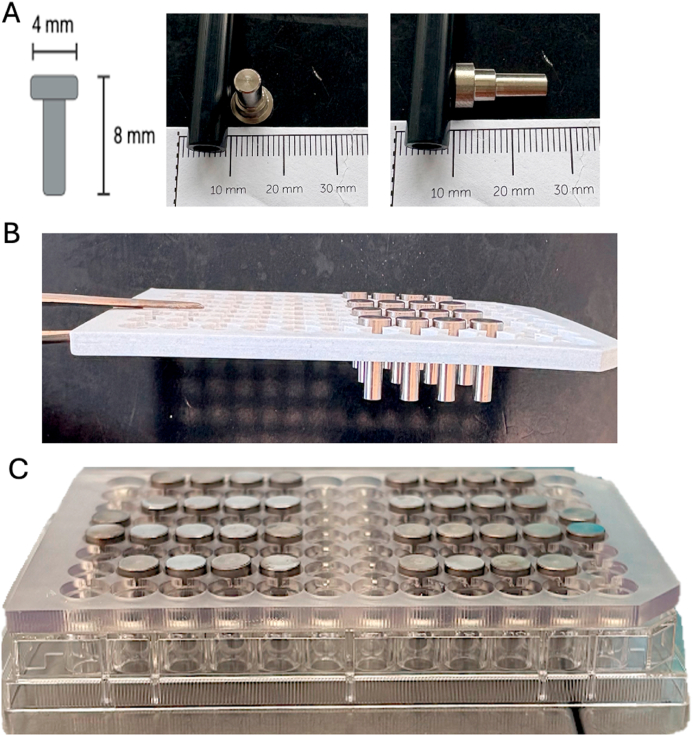
Photography of Titanium Peg Model: A. View of Titanium Peg with dimensions, B. Holder with Titanium Pegs, C. Holder with Titanium Pegs on a 96-well plates.

To optimize the incubation conditions, the duration in latter experiments was modified by allowing the bacteria to adhere to the supports (pegs) for 8 h. After this initial adhesion phase, pegs were transferred into new wells containing fresh, appropriate medium. This modified procedure is further named “8–72 h” method.

i. Disk model:

Bacterial adhesion was assessed using titanium discs measuring 12 mm in diameter and 5 mm in height (Fig. 1) (ACNIS, France), previously shotpeened by CRITT-MDTS (Charleville-Mézières, France). Each disc was placed at the bottom of a well in a 24-well plate, and 500 μL of the bacterial inoculum was added to each well, allowing contact between the disc surface and the bacterial suspension.

ii. Peg model:

In parallel, titanium pegs (4 mm in diameter × 8 mm in height) were used following the same surface treatment (Fig. 2A). The pegs were mounted on a specialized support (Fig. 2B) compatible with 96-well plates, allowing them to be suspended in the wells and oartly immersed in medium (Fig. 2C). For each well, 150 μL of the bacterial inoculum was added, enabling bacteria to adhere to the surface of the pegs during incubation.

### 2.3 Bacterial enumeration

i. Disc

After incubation periods of 24, 72, 96, or 144 h, the samples discs were washed by total immersion in small container with 5 mL of media used for inoculation to remove non-adherent bacteria, and then transferred to tubes containing 2 mL of PBS. To detach biofilm-embedded bacteria, the tubes were placed in an ultrasonic (*VWR*) bath (40 kHz) for 5 min.

ii. Peg

After incubation periods of 72 h, the pegs were transferred and partially immersed in a 96-well plates, each well containing 200 μL of fresh medium, to remove non-adherent bacteria. Then, pegs were transferred to tubes containing 1 mL of PBS. To detach biofilm-embedded bacteria, the tubes were placed in an ultrasonic (*VWR*) bath (40 kHz) for 5 min.

Subsequently, for both Discs and Pegs, 100 μL from serial dilutions (PBS 1× ThermoFisher) of the suspensions were plated on TSA and incubated ON at 37 °C to quantify the adherent bacteria. Simultaneously, to evaluate the planktonic bacteria, serial dilutions of the supernatants present in the well were plated on TSA plates.

Briefly dilutions were seeded on TSA plates using automatic seeder EasySpiral (Interscience, Saint-Nom-la-Bretèche, France). After 24 h of incubation, the number of recovered colony-forming units (CFU) was determined using automatic counter SCAN 1200 (Interscience, Saint-Nom-la-Bretèche, France) and the quantity of live adherent bacteria was determined as follows: CFU/mL normalized (per mm^2^). At least three independent experiments were performed with *S. aureus* strain.

### 2.4 Bacterial biomass quantification

As previously described, biofilm biomass was evaluated using crystal violet staining (Reffuveille et al., 2014). Briefly, discs were gently washed one time with appropriate medium, then discs were immersed in tubes containing equivalent medium volume for a few seconds, after that 500 μL of 0.18 % Crystal violet was added to each well. Plates were incubated for 20 min in the dark at room temperature. Discs were then washed by immersion in 3 separate wells containing 1 mL of water. After washing, 500 μL of 95 % ethanol was added to each wel.

For peg models, the pegs were briefly immersed in a well with an amount of 200 μL of media before being placed in a well with 200 μL of 0.18 % Crystal violet. Plates were incubated for 20 min in the dark at room temperature. Pegs were then washed, by dipping three time with in distilled water. After washing, 200 μL of 95 % ethanol was added to each well. The absorbance at 595 nm was measured to evaluate the amount of biofilm biomass recovered in each well, which is proportional to the absorbance value. The absorbance at 595 nm was measured to evaluate the amount of biofilm biomass, which is proportional to the absorbance value. Blank value have been soustracted to the absorbance of each well.

### 2.5 Confocal microscopy

After incubation, the pegs were washed once in PBS (1×) and stained with SYTO-9™ and propidium iodide diluted in 0,9 % NaCl at 1 μM and 20 μM respectively to label live and damaged or dead bacteria.

After 30 min of incubation in the dark at room temperature, each titanium peg was washed one time with PBS. A new well was filled with PBS and the peg was dipped in it. After that it was placed in a Krystal 96-well plate with glass bottom (Porvair, United Kingdoms) before observation with confocal laser scanning microscopy (CLSM) (LSM 710 NLO, Zeiss, Germany). Fluorochromes were imaged and their volume was quantified using IMARIS software (Imaris V8.9).

### 2.6 Stastistical analysis

The statistical significance of the results was assessed using the exact 2-tailed non-parametric Wilcoxon-Mann-Whitney test of GraphPad Prism (v8.0.1) software. Differences were considered significant at *p* < 0.05. Fold-increase or fold-decrease refers to the comparison of average difference for each run.

## 3 Results and discussion

### 3.1 Selection of environmental parameters for *S. aureus* biofilm study

**Fig. 3:**
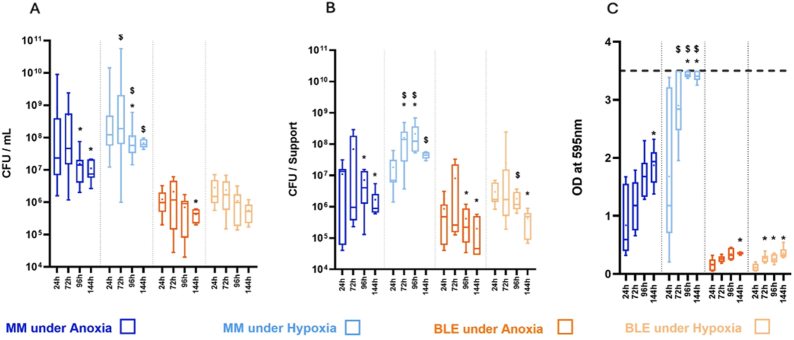
Kinetics of S. aureus planktonic, adherent development on titanium discs and biomass production: A) Planktonic part in CFU/mL; B) Adherent part in CFU/mL; C) Biomass measurement in absorbance at 595 nm. Experiments were done using a model of growing bacteria on a raw titanium disc in a 24 wells plates. Results represent the number of viable bacteria n = 3 to 6 (the boxes represent the maximum and minimum values of the bacteria count, the square shows the mean): Dark blue: MM and anoxic condition; Light blue: MM and hypoxic condition; Dark orange: BLE and anoxic condition; Light orange: BLE and hypoxic condition. ∗ indicates a significant difference compared to 24 h. $ indicates a significant difference compared to the same time under anoxic conditions. MM and BLE are significantly different except for adherent bacteria at 72 h under anoxia. Difference was considered significant for p < 0.05. The dotted line in C represents the saturation level. (For interpretation of the references to colour in this figure legend, the reader is referred to the Web version of this article.)

To develop a PJI model that closely mimics physiological conditions, we first investigated three key parameters influencing *S. aureus* biofilm formation: nutrient availability (MM and BLE), oxygen concentration (anoxia and hypoxia) and incubation duration (0 h–144 h). Experiments were conducted using the disk model. Fig. 3 is organized according to three key factors: (1) medium composition, (2) Dioxygen concetration, and (3) incubation time. For each factor, we evaluate trends across planktonic growth, adhesion, and biofilm biomass.

i. Effect of Medium Composition

Bone also serves as a major magnesium reservoir, where it supports mineralization, osteogenic differentiation, and structural integrity (Dutta et Layton 2024). Moreover, the bone microenvironment is often limited in glucose and amino acids, imposing metabolic stress on invading bacteria and determining their survival strategies [16].

A nutrient-minimum medium (MM) consistently supported greater bacterial proliferation and biomass accumulation compared to the nutrient-limited 10.13039/501100010771BLE medium (Fig. 3). 10.13039/501100010771BLE supported much lower planktonic bacteria than MM (e.g., 2.12 × 10^6^ 10.13039/100026585CFU/mL *vs* 5.07 × 10^8^ 10.13039/100026585CFU/mL at 72 h under anoxia, respectively). Similarly, biofilm biomass in MM reached absorbance values of 1.9 at 144 h, compared to a maximum of 0.36 in BLE. Adherent cell counts were also significantly higher in MM compared to BLE (6.90 × 10^7^ CFU/mL *vs* 8.04 × 10^6^ CFU/mL at 72 h under anoxia, respectively). Overall, MM led to significantly higher planktonic growth, adherent bacteria growth, and matrix formation, at each time point under anoxia.

However, the BLE medium imposed metabolic constraints that favored bacterial adhesion over proliferation. For example, when calculating the percentage of bacteria present in the adherent part out of the total bacteria present, 50.20 % of the bacterial population was adherent in BLE under anoxia, compared to only 5.47 % in MM at 72 h. This shift supports previous findings that nutrient limitation promotes transition to a biofilm-forming phenotype [26], and Mg^2+^ enhances *S. aureus* adhesion [27].

ii. Effect of Atmospheric Condition (Hypoxia vs. Anoxia)

Bone tissue, particularly the marrow, is markedly hypoxic, with oxygen levels typically ranging from <1 % to 6 %, and around 2 % in many areas [28], while *S. aureus* is able to exploit alternative metabolic pathways such as shunting pyruvate into the butanediol pathway to maintain redox balance during anaerobic growth or fermentation [29].

The present results showed that, under hypoxia, both planktonic and adherent populations peaked higher and declined later than under anoxia (Fig. 3). In every media tested in the present study, *S. aureus* showed better planktonic growth under hypoxia (2.5 % O_2_) than under anoxia (0 % O_2_).

Adhesion was also favored under hypoxia. Using MM at 72 h timepoint, the amount of adhering bacteria were 2.3 times higher than under anoxia. Likely to the percentage of adhering bacteria compared to the total quantity of bacteria, only 5.47 % were under this conformation under anoxia compared to 37.54 % under hypoxia. Lastly, the total biofilm biomass formation in MM under hypoxia was more robust compared to the anoxia condition, achieving saturation levels earlier (by 72 h). The low biomass formation in BLE did not allow to detect such a clear difference between both atmospheres regarding this medium.

These findings align with reports highlighting oxygen gradients as a major determinant of *S. aureus* biofilm development [30]. Oxygen levels modulate biofilm dynamics: hypoxia can promote biofilm formation by upregulating adhesion and matrix synthesis genes [31] and is more relevant to PJI, where localized hypoxia influences bacterial persistence and biofilm dynamics [32].

iii. Effect of Incubation Time

Overall, bacterial growth and adhesion peaked around 72 h in both media and under both atmospheric conditions. For planktonic populations, growth plateaued after 72 h, while adherent cells and biofilm biomass began declining by 96 h, continuing through 144 h.

In MM, the decline phase began after 72 h, with a 50-fold drop and a 30-fold drop under anoxia and hypoxia respectively at 96 h, after reaching the maximum values at 72 h. In BLE, the decline seemed to start between 72 h and 144 h, and is particularly strong on adherent bacteria with CFU counts decreasing significantly below baseline (24 h) by 144 h under bot atmospheres. These declines may be attributed to nutrient depletion, metabolic shifts, and dispersal mechanisms [32,33].

iv. Overall analysis

To sum up, the dynamics of *S. aureus* growth and biofilm formation varied notably depending on the medium, oxygen availability, and incubation time. Moderatly nutrient-supplied medium (MM) promoted greater planktonic growth, adhesion, and biomass accumulation compared to the nutrient-poor BLE medium. Hypoxic conditions (2.5 % O_2_) consistently favored bacterial proliferation and adhesion over anoxia, reflecting *S. aureus* ability to exploit alternative metabolic pathways. Over time, planktonic and adherent populations increased up to 72 h before declining, while biomass continued to accumulate over the 144 h of the tests.

Together, these features highlight that nutrient availability and oxygen concentration jointly modulate biofilm behavior, with 72 h emerging as an optimal time point for biofilm analysis before dispersal begins. This timeframe aligns with established biphasic patterns of biofilm development and underscores the necessity of selecting an adapted incubation period for experimental analyses for *in vitro* biofilm models [20].

These first results led to the validation of these two key parameters of this innovative model. Hypoxia was choosen regarding its biological relevance and how influencial it can be toward bacterial behavior. Regarding incubation time, 72 h was determined as optimal, as it corresponded to peak biofilm formation before the onset of decline, allowing to consistently obtain observable biofilm in a reasonable amount of time. In any case, we were able to observe that whatever the medium, the impact of oxygen and incubation time showed similar trends. To simulate the nutrient limitations characteristic of poorly vascularized bone post-surgery [34], BLE medium were developped in a previous study ([16] Although bacterial growth and biomass production in BLE were significantly lower than in MM, the proportion of adherent bacteria was markedly higher, consistently with other findings [28,35,36].

### 3.2 Optimisation of culture medium

Given the study focus on biofilms and adherent bacteria, BLE was initially chosen for its ability to promote a high proportion of biofilm-associated cells. However, the extreme nutrient limitations of BLE made biofilm visualization challenging in CV assay. To overcome this, we developed BLE+, a slightly enriched version of BLE and still reflecting the nutrient conditions present in bone tissue (*Karner* et al. *2018*).

According to the review by American Diabetes Association (2022) normal fasting plasma glucose levels in healthy adults range between 70 and 100 mg/dL (0.7–1 g/L). As glucose also plays a crucial role in biofilm formation in *S. aureus* because even at low concentrations, glucose can strongly influence biofilm development by inhibiting the P3 promoter of the *Agr* system, which negatively regulates biofilm formation [1]. Concentrations of less than or equal to 1 mg/mL are found in the bone environment, whereas synovial fluid is more variable and richer than bone during an infection ([37]; Dawan et Ahn 2022). Here, the chosen concentration is in the lower range of the serum concentration (80 mg/dL so 0.8 g/L).

The total protein concentration in healthy synovial fluid is approximately 25 mg/mL, but only a small portion is available for bacterial metabolism and in bone site (CRIOAC2015; [37]; Dawan et Ahn 2022)). Here, a concentration of 1 mg/mL of casaminoacids has been choosen [37].

**Fig. 4:**
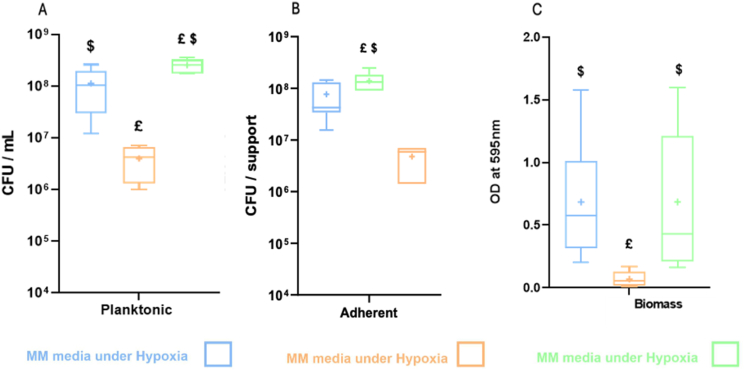
Comparison between BLE+, BLE, and MM on bacterial growth: a) Planktonic bacteria; b) Adherent bacteria; c) Biofilm biomass on disks. Experiments were done using a model of growing bacteria on a raw titanium disc in a 24 wells plates. (the boxes represent the maximum and minimum values of the bacteria count): Light blue: MM; Light orange: BLE; Light green: BLE+. £ indicates a significative difference compare to MM. $ indidcates a signifactive difference compare to BLE. Difference was considered significant for p < 0.05. (For interpretation of the references to colour in this figure legend, the reader is referred to the Web version of this article.)

The impact of BLE + after 72 h under hypoxic conditions was compared to MM and BLE (Fig. 4). Bacterial counts were the highest in BLE+, intermediate in MM, and the lowest in BLE. This trend was consistently observed in both planktonic bacteria (Figs. 4A and 1.10 × 10^8^ CFU/mL, 3.99 × 10^6^ CFU/mL, and 2.5 × 10^8^ CFU/mL for MM, BLE and BLE+, respectively) and adherent bacteria (Figs. 3B and 7.70 × 10^7^ CFU/mL, 4.79 × 10^6^ CFU/mL and 1.39 × 10^8^ CFU/mL for MM, BLE and BLE+, respectively). When comparing the proportion of adhering bacteria in the different media, MM showed the highest percentage with 40.26 % followed by BLE with 47.19 % and BLE+ with 31.84 %. However, no significant difference in biomass formation (Fig. 4C) was observed between MM and BLE + but both were significantely differente from BLE biomass.

Developing BLE + medium, with five times higher glucose and increased amino acid concentrations than BLE, improved bacterial adhesion and signal detection while maintaining physiological relevance. These refinements enabled the development of a more robust and biologically relevant model for studying *S. aureus* biofilms in the context of PJI. Despite its nutrient depletion, which maintains stress conditions and promotes bacterial adherence, it still allows the threshold values to be surpassed. This validates its relevancy and suitability.

### 3.3 Implementing a new biofilm support: the titanium pegs

In the presence of synovial fluid or components of bone and joint tissues, *S. aureus* rapidly forms large, suspended aggregates [38]. Bacterial aggregates, such as those formed by *S. aureus*, may indeed settle onto implant surfaces due to gravity, especially if the aggregates are large or if the fluid is relatively quiescent [39]. Due to gravity, these aggregates readily settle on implant materials and can initiate biofilm formation. Studies on titanium and other orthopedic surfaces have shown that aggregates adhere more robustly than planktonic cells, particularly in surface grooves under physiologically relevant conditions [38,40]. Planktonic cells adhere only weakly to smooth surfaces and are easily removed by fluid shear stress [40,41]. In contrast, bacterial aggregates secrete ECM components, such as Fbps, eDNA, and polysaccharides, that act as biological glue, enhancing adhesion to metal oxides [41,42].

Therefore, our model aims to highlight the difference between a system allowing the formation of aggregates that sediment (Disk model) and a model allowing the formation of aggregates without sedimentation (Peg model) [43,44].

**Fig. 5:**
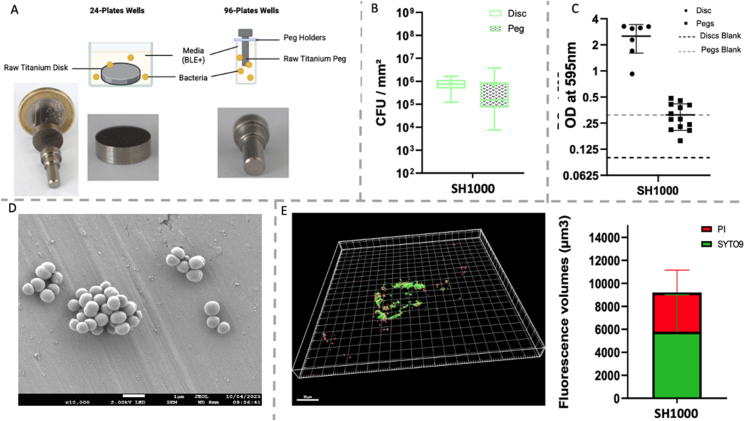
Transfer of the disc model to a new technology, the peg. All this figure presents results obtained with SH1000 strain: A) Diagram of technology transfer and illustrative photographs. B) Quantity of adherent bacteria on each support (results are showed by CFU per mm^2^). Experiment was performed at least three times with duplicate. C) Absorbance of the total biomass (raw data without blank correction) labelled with Crystal Violet and read at 595 nm. Experiments was performed at least two times with triplicate for disc and three times for pegs. D) Observation of SH1000 biofilm using scanning electron microscopy (SEM) on pegs surfaces (×5000). E) Observation and measurement of SH1000 biofilm fluorescence levels using confocal microscopy 3D representation of biofilm using IMARIS software. Scale barre corresponds to 50 μM. (For interpretation of the references to colour in this figure legend, the reader is referred to the Web version of this article.)

To prevent planktonic bacterial aggregates from settling on titanium discs and interfering with biofilm development, we designed a titanium peg model supported by a holder positioned beneath the cover (Fig. 5A). This configuration with suspended pegs in culture medium aims to minimize sedimentation.

Comparative analysis revealed that titanium discs harbored a significantly higher density of bacteria (9.91 × 10^5^ CFU/mm^2^) than the pegs (3.5 × 10^5^ CFU/mm^2^) (Fig. 5B). Although to a lesser extent than on disks, the biofilm biomass is still detectable on pegs (Fig. 5C). Scanning Electronic Microscopy (SEM) and Confocal Laser Scanning Microscopy of the pegs showed adherent bacterial aggregates (Fig. 5D and E) approximately 25 μm thick, predominantly composed of live bacteria, accounting for an average of 62 % of live bacteria in the total fluorescent volume (Fig. 5E). Despite fewer aggregates on the pegs, their viability underlines the model suitability for further analyses.

In our experiments, adherent bacteria were present on both disc and peg models. Peg model minimized interaction between planktonic aggregates and biofilm formed on the surface thanks to its suspension from the lid, avoiding aggregates to sediment on titanium support.

The peg model not only reduces aggregate interference but also facilitates more accurate observation of surface-attached biofilms: it allows for direct biofilm visualization under confocal microscopy. Briefly, after incubation of 72 h, pegs are transferred to a new plate for live and dead bacteria visualization without having to turn the structure upside down like in the disc model, thus avoiding an additional step that could alter the observation. Moreover this feature make it easier to assess structural characteristics and spatial distribution of bacteria.

Despite some limitations in the sensitivity of standard quantification techniques like CV staining, this model remains a robust platform for biofilm analysis. Future improvements in detection sensitivity could further enhance the model accuracy and utility. Switching to the peg model allowed to limit the presence of planktonic aggregates, which are structurally and functionally distinct from biofilms. *S. aureus* is known to form large planktonic aggregates during the early exponential growth phase, with sizes reaching up to 0.5 mm depending on the strain and environmental conditions [45]. These aggregates, which can be promoted by osmotic stress and low oxygen tension, exhibit increased antibiotic tolerance, similar to biofilms, but differ in their organization and metabolic behavior. Under hypoxic conditions, oxygen limitation further favors planktonic aggregate formation, potentially impeding biofilm development by stimulating bacterial metabolism and accelerating nutrient depletion [12,43,46]. By avoiding these adverse effects, the peg model offers a more controlled environment for studying biofilm formation.

However, in our *in vitro* model, the presence of planktonic bacteria during the initial 72 h of culture could substantially influence biofilm development, potentially leading to characteristics that do not accurately reflect *in vivo* conditions.

### 3.4 Elimination of stationary planktonic bacteria and restocking of nutrients

Archer K·N et al. [47] demonstrate that planktonic forms of *S. aureus* are rapidly replaced by biofilms or variants adapted to intracellular persistence during osteoarticular infections. As part of further improving biofilm study protocol and avoiding the phenomenon of stationary planktonic bacteria, a medium renewal was introduced after 8 h of incubation over a total incubation period of 72 h as defined in 2. b (M&M). This adjustment aimed to reduce the influence of planktonic bacteria on biofilm formation and to mimic a new nutrient supply.

Notably, the concentration of planktonic bacteria per milliliter was significantly reduced after 72 h with medium renewal compared to unrenewed medium (4.8 × 10^6^ CFU/mL *vs* 3.5 × 10^8^ CFU/mL, respectively) indicating a low bacterial dispersion from the biofilm to the planktonic phase.

**Fig. 6:**
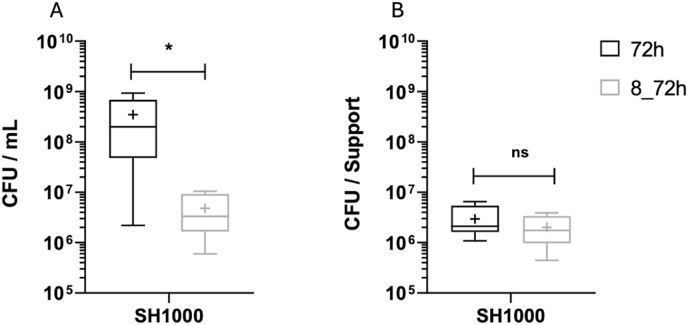
Impact of medium renewal after 8 h incubation on SH1000 strains. Planktonic bacteria (5. A) in CFU per mL and on adherent bacteria (5. B) in CFU per support. Boxes represent the maximum and minimum values of the bacteria count after 72 h of incubation in BLE+. The cross shows the mean value for each boxes. Experiments made 3 times with duplicates. Difference was considered significant for p < 0.05.

However, no significant difference was observed in the enumeration of adherent bacteria between protocols with and without medium renewal (Fig. 6).

By removing planktonic bacteria, we aimed to allow the biofilm to develop independently, more closely mimicking conditions observed in the human body [42,48,49]. By excluding bacterial aggregates from this *in vitro* model the primary objective was to specifically investigate the dynamics of adhesion and biofilm development on a defined abiotic surface (pegs). *In vitro* aggregates differ from those formed *in vivo*; therefore, they are not truly representative, can interfere with the natural biofilm balance, and are thus undesirable in the model[50].)On pegs, the presence of a thin biofilm resulting exclusively from the adhesion of metabolically active bacteria was observed, making it more representative of real conditions and therefore more relevant. Medium renewal decrease the number of planktonic bacteria without restoring planktonic growth to initial levels after 72 h. Our hypothesis is that dispersed bacteria transitioning from biofilm to planktonic states exhibited reduced proliferation, reaching a new equilibrium at 72 h. This growth reduction aligns with findings from previous studies on *Pseudomonas aeruginosa*, where dispersed populations displayed slower growth rates compared to initial planktonic cultures [51]. Both biofilm-associated and dispersed bacteria exhibited comparable growth rates, suggesting a physiological shift upon biofilm dispersal [52].

### 3.5 Final model

**Fig. 7:**
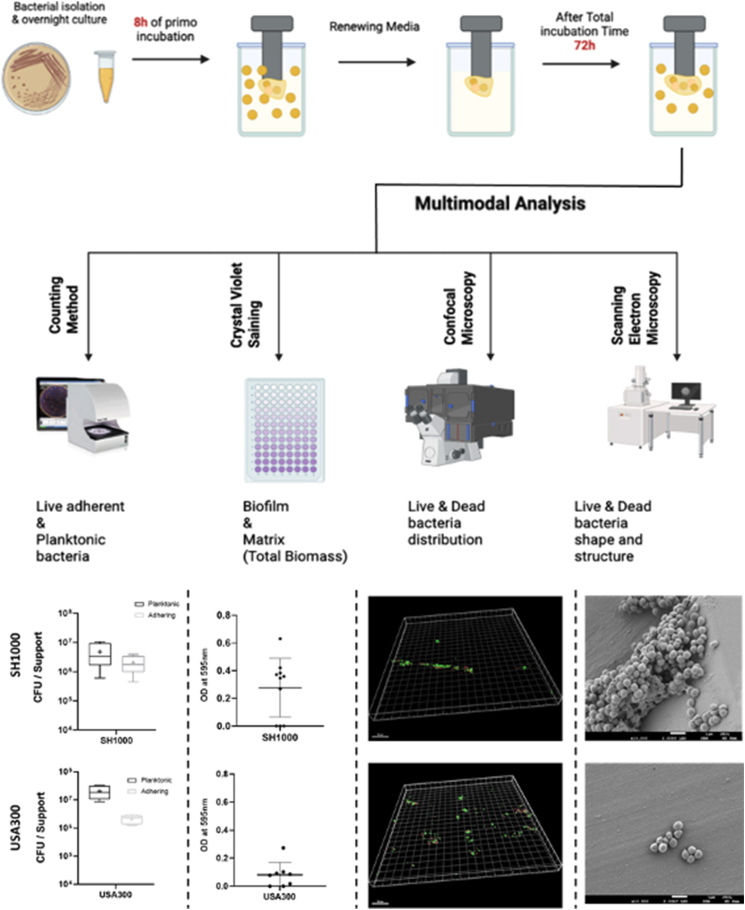
Summary diagram of the InVitrOs model comparing two strains, on the top the MSSA strain (SH1000) and at the bottom the MRSA strain (USA300) to characterize the strain. From the left to the right: First diagram shows planktonic and adherent bacteria quantification (black box and grey box respectively), the cross shows the mean value. Secondly, absorbance of the total biomass (blank-corrected) labelled with Crystal Violet and read at 595 nm. Third one, biofilm fluorescence observation using confocal microscopy 3D representation of biofilm using IMARIS software. Scale barre corresponds to 50 μM. Forth one, biofilm observation using scanning electron microscopy (SEM) (×10000) on pegs surfaces Experiments was performed at least two times with duplicates. (For interpretation of the references to colour in this figure legend, the reader is referred to the Web version of this article.)

To further challenge our model, another strain was tested, USA300, a methicillin-resistant *S. aureus* (MRSA) strain known for forming biofilms that differs from SH1000 strain [16] (Fig. 7). As expected, bacteria were detected under both planktonic (2.05 × 10^7^ CFU/mL) and adherent (4.12 × 10^6^ CFU/mm^2^) phases. Cristal violet staining (after blank correction) revealed that USA300 produced a lower biomass SH1000. While the biofilm formation in the model can be observed by this method, the low and even below threshold values of OD (three samples for SH1000 and two samples for USA300 below threshold) demonstrate the variability linked to this technique. This alert on the necessity to repeat as many cristal violet tests as possible and to interprete results with caution.

The biofilm structure also differed, as revealed by CLSM and SEM imaging. While SH1000 formed dense biofilms, USA300 biofilms appeared more dispersed, with smaller aggregates spread across the surface. This pattern matches with previous data on the USA300 strain, which tends to produce a protein-rich matrix due to *ica* gene repression [47]. Fluorescence quantification supported this observation, with a declining tendency when looking at the total volume (6050.1 μm^3^ vs. 9206.4 μm^3^ for USA300 and SH1000 respectively) but a slightly higher proportion of live cells in USA300 (70 % vs. 61.7 %).

Thanks to the model, it is possible to discriminate the two strains studied on the basis of the 4 parameters: planktonic and adherent bacteria quantification, biomass absorbance, live/dead proportion labelling (CLSM) and biofilm structure (SEM) tested.

Altogether, these results show that the model can reflect known strain-specific biofilm behaviors found in the literature [16], reinforcing its relevancy to study diverse biofilm phenotypes under controlled conditions.

USA300 is known to use surface proteins, such as FnBP-A/FnBP-B, autolysin Atl and eDNA to form PIA-independent biofilms. These biofilms are therefore more heterogeneous and less adherent and completely different from those of MSSA (like SH1000) strains which was observed in this study [16].

Thus, we were able to characterize two strains according to the four parameters mentioned earlier. The model developed in this study appears to provide strain dependant response, but its robustness remains to be strengthened by a wider range of strains to be tested. However this model open up the possibility of differentiating strains based on the different aspects provided by this model.

## 4 Conclusion

Lead by the hope to reduce the use of animal testings, developing a physiologically relevant *in vitro* model presents significant challenges due to the need to precisely control multiple parameters. To address this objective, we designed a model that appears robust, reproducible, relevant, practical, and easy to use. This effort led to the development of the *InVitrOs* model, which operates under well-defined conditions: 72 h of incubation of a suspended titanium support (pegs) under hypoxic conditions, with media renewal after 8 h. The model was specifically designed to allow bacterial enumeration (planktonic and adherent), biomass quantification, biofilm structure observation and proportion of live and dead bacteria analysis. All these techniques being possible within a single plate, this innovative model could enable routine analysis in a 96-well format, particularly for studying biofilm characteristics of PJIs strains. Especially, as biofilm is a persistence mechanism that is (nearly) unstudied in diagnostics, this model could be a real innovation in the clinic, but also for antimicrobial testing destined for bone. Additionally, the model allows the investigation after 72 h growth, offering a good compromise between a relevant incubation delay and a reasonable time to obtain a potential diagnosis. Design optimizations can be implemented to enhance bacterial adhesion, since it only results from actively adhering bacteria. The peg grip can also be enhanced for greater practicality.

Further experiments will focus on characterizing others strains and particularly clinical strain of *S. aureus* to highlight their behavior and see the characteristics of each strain using our new model (InVitrOs). Eight strains will be tested, including four MRSA and four MSSA strains, with differential characterization of the strains based on various parameters (microbiological, structural, and phenotypic), leading to a future publication.

Using this model for antibiofilm candidate screening would represent a significant advance in optimizing biofilm-targeted therapies. This model provides a robust platform for characterizing biofilms from clinical *S. aureus* strains, paving the way for further research into biofilm-related infections.

## CRediT authorship contribution statement

**Yasser Dghoughi:** Writing – review & editing, Writing – original draft, Methodology, Investigation, Formal analysis, Data curation. **Jennifer Varin-Simon:** Writing – review & editing, Methodology, Investigation. **Sophie C. Gangloff:** Writing – review & editing, Validation, Supervision, Funding acquisition. **Marius Colin:** Writing – review & editing, Validation, Supervision. **Fany Reffuveille:** Writing – review & editing, Visualization, Validation, Supervision, Resources, Project administration, Funding acquisition, Formal analysis, Data curation, Conceptualization.

## Funding

The Ph.D. fellowship (InVitrOS program) of YD was co-funded by Region Grand Est, Strasbourg, France and the university URCA, Reims, France. The project was supported by BIOScreenTarget (PRC) - ANR-23-CE19-0012-03 and Institut Carnot MICA (InvitrOs project).

## Declaration of competing interest

The authors declare that they have no competing interests.

## Data Availability

Data will be made available on request.
